# Liver Remodeling on CT Examination in Patients with HCV Compensated Cirrhosis Who Achieved Sustained Virological Response after Direct-Acting Antivirals Treatment

**DOI:** 10.3390/medicina56040171

**Published:** 2020-04-10

**Authors:** Florin Mihai, Anca Trifan, Carol Stanciu, Ana Maria Singeap, Bogdan Cucuteanu, Corina Lupascu Ursulescu, Corina Pop, Irina Girleanu, Tudor Cuciureanu, Dragos Negru, Camelia Cojocariu

**Affiliations:** 1 “Grigore T. Popa”, University of Medicine and Pharmacy, 700115 Iasi, Romania; florinmihai77@gmail.com (F.M.); bogdan.cucuteanu@gmail.com (B.C.); corina.ursulescu@gmail.com (C.L.U.); gilda_iri25@yahoo.com (I.G.); drcuciureanutudor@gmail.com (T.C.); draneg@gmail.com (D.N.); cameliacojocariu@yahoo.com (C.C.); 2 Department of Radiology, “St. Spiridon” Emergency Hospital, 700111 Iasi, Romania; 3 Institute of Gastroenterology and Hepatology, “St. Spiridon” Emergency Hospital, 700111 Iasi, Romania; stanciucarol@yahoo.com; 4 Gastroenterology Department, Emergency University Hospital, “Carol Davila” University of Medicine and Pharmacy, 050474 Bucharest, Romania; cora.pop@gmail.com

**Keywords:** liver cirrhosis, computed tomography, liver fibrosis, hepatitis C virus

## Abstract

*Aims:* The purpose of this study was to assess the changes in hepatic morphology evaluated by computed tomography (CT) examination in patients with hepatitis C virus (HCV)-related compensated cirrhosis who achieved sustained virologic response (SVR) after direct-acting antivirals (DAAs) treatment. *Methods:* CT examination was performed in 56 patients with HCV-related compensated cirrhosis before and within 6–18 months after the treatment with Ombitasvir/Paritaprevir/ritonavir + Dasabuvir. The liver CT changes were assessed by measuring liver volume, caudate-right lobe ratio (C/RL), hepatic vessels diameters, periportal widening space, and right posterior notch. Portal trunk, splenic and superior mesenteric vein diameters, as well as spleen volume were assessed as part of portal hypertension. *Results:* Right hepatic vein diameter was significantly wider after treatment (median: 8.12 mm; IQR: 4.20) than before treatment (median: 6.36 mm; IQR: 3.94) z = −3.894; *p* < 0.001. The liver volume was significantly higher prior to the treatment (median: 1786.77 mm^3^; IQR: 879.23) than after treatment (median: 1716.44 mm^3^; IQR: 840.50), z = −1.970; *p* = 0.049. Splenic volume was considerably higher before treatment (median: 564.79 mm^3^; IQR: 342.54) than after (median: 474.45 mm^3^; IQR: 330.00), z = −2.500; *p* = 0.012. The other parameters, such as C/RL, periportal space widening, and right hepatic notch showed no significant changes. *Conclusions:* SVR in patients with HCV-related compensated cirrhosis treated with DAAs is associated with some improvements of hepatic morphology detectable by CT, the most constant being the increase of right hepatic vein diameter.

## 1. Introduction

Liver cirrhosis is a major cause of morbidity and mortality worldwide and is currently the 11th most common cause of death on a global scale, accounting for 3.5% of all deaths [1]. Worldwide, one of the main causes of cirrhosis is the infection with hepatitis C virus (HCV) [2]. Direct acting antivirals (DAAs) introduced in 2014 have represented a revolution for the treatment of patients with HCV due to high sustained virologic response (SVR) rate (95–100% in genotype 1) and excellent tolerability [3,4]. Despite the proven SVR, there is still a risk of developing HCC that justifies careful monitoring of these patients [5].

Traditionally, cirrhosis was considered an irreversible end stage of liver disease, mostly due to lack of treatment possibilities. Nowadays, this irreversibility is no longer considered a “dogma” as liver fibrosis is potentially reversible on the condition that the trigger is removed [6]. There is evidence that patients who achieve SVR are less likely to develop liver-related complications, due to the regression of fibrosis after HCV eradication [7]. Several non-invasive methods for assessing liver fibrosis confirmed fibrosis regression in patients with SVR [8,9,10].

Liver cirrhosis is characterized by significant parenchymal and vascular architecture change with formation of septae and regenerative nodule. HCV-related cirrhosis is a consequence of an ongoing liver injury by the hepatitis C virus. Among the most important factors involved in regression of hepatic fibrosis is the elimination of the primary cause of the chronic hepatic injury. Thus, elimination of HCV achieved by the DAA treatment is the cardinal condition for regression of liver cirrhosis [11].

Sectional imaging techniques, such as computed tomography (CT), magnetic resonance imaging (MRI), and ultrasonography (US) have been used in cirrhosis diagnosis and staging with variable rates of success. These techniques have different independent predictive signs for the diagnosis of liver cirrhosis [12,13]. US is the most accessible and most used imaging method for the evaluation of patients with chronic liver disease; liver cirrhosis diagnosis using US has an accuracy, sensitivity, and specificity of 64–79%, 52–69%, and 74–89%, respectively [12,14]. MRI sensitivity and specificity in liver cirrhosis are 87% and 54%, respectively, similar to those of CT [12].

Abdominal CT scans are frequently performed in clinical studies involving cirrhotic patients due to its ability to diagnose and rapidly stage HCC following contrast administration. For this reason, CT is usually used in patients with HCV-related cirrhosis prior being involved in the therapy with DAAs in order to exclude HCC (suspected at US) or other malignancy which is a contraindication to this treatment. Additionally, CT can diagnose liver fibrosis in early, precirrhotic stage. It is also useful to evaluate the extrahepatic complications of cirrhosis, such as portal hypertension and its effect on the abdominal organs.

The classical imaging features of cirrhosis identifiable by CT scans include hypertrophy of the caudate lobe, as the most characteristic morphologic sign of the disease and, in more advanced stages, hypertrophy of the lateral segments of the left lobe (II and III) with atrophy of the medial segment (IV) and of the posterior segments (VI and VII) of the right lobe [14]. Volume changes in the left lobe medial segment and right lobe segments lead to the widening of gallbladder fossa and the enlargement of central periportal space (defined as the distance between the anterior wall of the right portal vein and the posterior edge of the medial segment of the left hepatic lobe, easy to assess with a cut-off value of 10 mm) [15,16]. Alteration of the caudate and right lobe morphology results in the presence of the right hepatic posterior notch sign, representing the functional lateral boundary of the hypertrophied caudate lobe and can be used as a simple and specific sign of cirrhosis [17]. However, CT overall diagnostic accuracy for liver cirrhosis is relatively low, with a sensitivity and specificity of 77–84% and 53–68%, respectively, as shown in a multicenter study [12].

The repair process that causes the formation of regenerative nodules also determines the compression of the central hepatic veins and decrease of hepatic veins diameters, as well as change in Doppler flow. A decreased right liver vein diameter below 7 mm should raise the suspicion of cirrhosis [18].

The caudate lobe hypertrophy is the foundation for development of imaging-based cirrhosis scores: first cirrhosis scoring based on axial imaging measurements was developed by Harbin et al. using the caudate-right lobe ratio (C/RL) by dividing the width of the transverse caudate lobe to the width of the transverse right lobe at bifurcation of portal vein [19]. A ratio ≥0.65 is a positive diagnostic indicator for cirrhosis, with 100% specificity, good sensitivity (84%), and accuracy (94%) [19]. A modified caudate-right lobe ratio (C/RL-m) was proposed, using the right portal vein bifurcation instead of main portal as lateral boundary with more accuracy for diagnosing cirrhosis and evaluating its clinical severity [20]. The latest imaging score for cirrhosis proposed by Huber et al. uses the combination of both morphological and vascular changes, dividing the sum of liver vein diameters by the C/RL-m [21].

The objective of this study was to identify changes in hepatic morphology that evoke reversibility of fibrosis using CT scans, in patients with HCV-related compensated cirrhosis who have achieved SVR following treatment with DAAs.

## 2. Methods

### 2.1. Study Design

This is a prospective study on patients with genotype 1 HCV-related compensated cirrhosis who have achieved SVR after treatment with DAAs (Ombitasvir/Paritaprevir/ritonavir+ Dasabuvir). Eligibility of enrolled patients was assessed following the criteria established by our National Health Insurance Agency and recommended by international guidelines: adult, treatment experienced or naïve patients with Child–Pugh class A cirrhosis assessed by Fibromax^®^ Biopredictive (cut-off of 0.71 for F4) or liver biopsy (F4 by METAVIR). Exclusion criteria were decompensated liver cirrhosis or evidence of hepatocellular carcinoma.

Every patient had a CT examination before treatment to exclude liver malignancy and to evaluate the liver morphology. After the treatment the imaging follow-up protocol included US at every 6 months.

A second CT examination was performed after the treatment, within 6 and 18 months following SVR achievement, to characterize nodular lesions or other parenchymal abnormalities detected by US.

All CT scans were anonymized and independently reviewed by three senior radiologists with experience in hepatobiliary radiology, blinded to all patient information. A fourth reader provided consensus in cases with disagreement in measurement.

In order to avoid errors related to the different section level in two different examinations, measurements were made after synchronization, as close to the same section level starting from anatomical landmarks.

The local Ethical Committee approved this study. Written informed consent was obtained from each patient and the study was conducted according to the Declaration of Helsinki.

### 2.2. Scanning Protocols

We used a Siemens Sensation^®^ 16 slice configuration CT scanner (Siemens AG Medical Solution, Erlangen, Germany). Our scanning protocol was optimized for detection of potential malignant liver lesion, according with CT/MRI Diagnostic LI-RADS^®^ recommendation for CT scanning protocol. This protocol includes anon-enhanced scan followed by i.v. administration of iodine-based contrast medium with tri-phase liver scan (arterial, portal and equilibrium phase). The contrast medium was administered in bolus with an injection rate of 3–5 mL/s. The arterial phase was acquired at 30–35 s (late arterial phase) and the venous phase at 75 s after contrast injection. The equilibrium phase was acquired at about 4 min after contrast injection. Patients were examined in the supine position, in post inspiratory apnea.

### 2.3. Data Analytic Strategy for Imaging Interpretation

The imaging data were evaluated for the following liver morphological changes: liver volume estimation using the following formula: volume = maximum dimension in cranio-caudal × latero-lateral × antero-posterior × 0.31 in cm^3^ [22]; values of C/RL and C/RL-m, which describe the width of the caudate lobe in proportion to the width of the right hepatic lobe; measurement of the hepatic vein diameters; measurement of the central periportal space widening; combination of hepatic vein diameter sums and caudate-right lobe ratio; assessment of right posterior hepatic notch variation; manifestations of portal hypertension: dilatation of portal system, including portal trunk, splenic vein, and superior mesenteric vein diameter and splenomegaly using the index value (product of cranio-caudal dimension, maximum size in axial plane and maximum thickness in axial plane) and the splenic volume (index value × 0.58 + 30) in cm^3^ [23].

### 2.4. Statistical Analysis

The statistical analysis started with the inspection of the continuous variables for assessing the normality of the distributions, including the normality of the distributions of the differences between the two repeated measurements. Since most of the differences did not meet the normality requirement, comparisons between the two sets of measurements, to see whether there were any changes in parameter levels, were performed with the Wilcoxon signed-rank test. A value of *p* < 0.05 was considered statistically significant.

## 3. Results

### 3.1. Patients

There were 56 patients (24 men and 32 women), mean age 57.78 ± 9.048 years (range 42–79).

**Figure 1:**
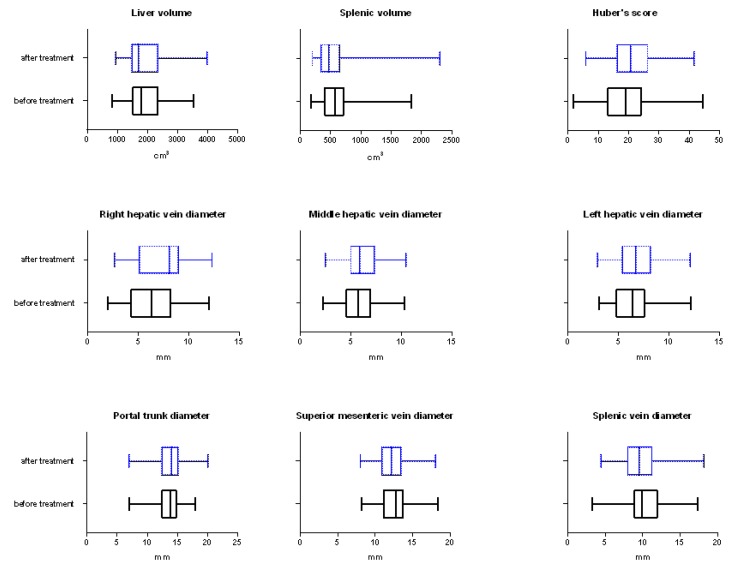
Boxplots of the main variables before and after the treatment comparison.

All the results are summarized in Table 1 and the graphical representation for the main variables is shown in Figure 1.

### 3.2. Hepatic Veins Diameters

Only the right hepatic vein diameter showed a statistically significant widening after treatment (median: 8.12 mm, IQR: 4.20), compared to the diameter recorded before treatment (median: 6.36 mm, IQR: 3.94), z = −3.894, *p* < 0.001. Both the middle and the left hepatic vein did not show significant changes in diameters after treatment. 

The overall scores for the vein diameters were statistically significantly higher after treatment (median: 21.44 mm, IQR: 9.06) compared to the measures taken prior to treatment (median: 19.29 mm, IQR: 9.52), z = −2.194, *p* = 0.028, a difference mainly accountable to the right hepatic vein differences.

**Figure 2:**
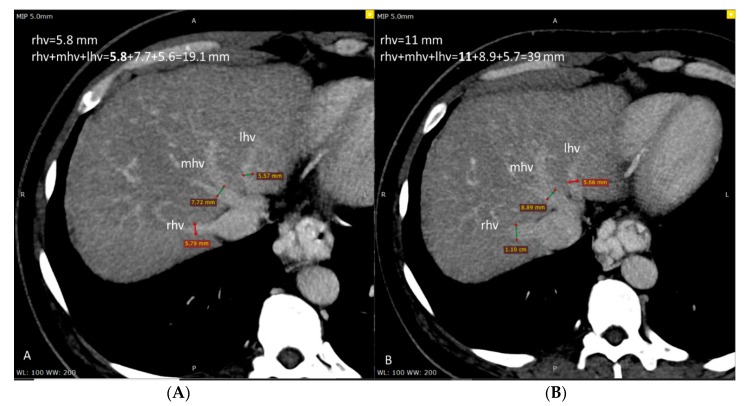
Computed tomography (CT) axial section at the level of hepatic veins before (**A**) and after (**B**) treatment. Only the right hepatic vein (rhv) showed significant widening after treatment (11 mm) compared to before (5.8 mm), while the middle hepatic vein (mhv) and left hepatic vein (lhv) showed no significant changes.

Illustrative changes are shown in Figure 2.

### 3.3. The Caudate-Right Lobe Ratio and Modified Caudate-Right Lobe Ratio

There were no statistically significant differences between the C/RL before (median: 0.65, IQR: 0.19) and after treatment (median: 0.65, IQR: 0.17), z = −1.283, *p* = 0.2, as well as between C/RL-m before (median: 0.98, IQR: 0.28) and after treatment (median: 0.99, IQR: 0.31), z = −0.597, *p* = 0.551.

### 3.4. Huber’s Score

**Figure 3:**
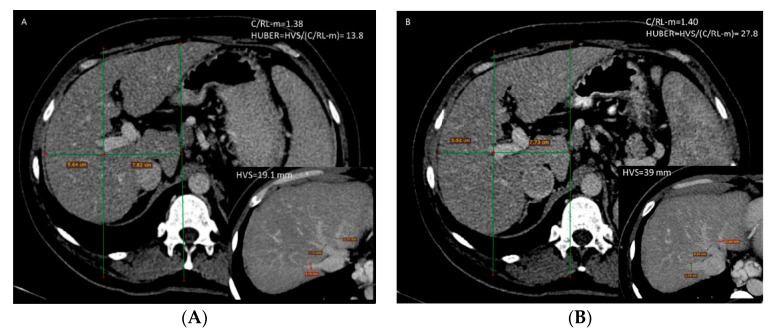
Huber’s score—before (**A**) and after (**B**) treatment in one patient. HVS: hepatic veins sum; C/RL m: caudate right lobe ratio modified.

Huber’s score before treatment (median: 19.16, IQR: 11.50) was significantly lower than after treatment (median: 20.58, IQR: 9.73), z = −2.106, *p* = 0.035, probably due to changes in hepatic vein diameters, because C/RL-m did not show meaningful changes (Figure 3).

### 3.5. Liver Volume

Liver volume of the patients prior to the treatment was significantly higher (median: 1786.77 cm^3^, IQR: 879.23) than after treatment (median: 1716.44 cm^3^, IQR: 840.50), z = −1.970, *p* = 0.049.

### 3.6. Central Periportal Space Widening

Results indicate there were no differences between central periportal space widening measured before (median: 11.85 mm, IQR: 5.75) and after treatment (median: 11.7 mm, IQR: 6.47), z = −0.368, *p* = 0.713.

### 3.7. Right Posterior Hepatic Notch Variation

There were no significant differences between right posterior hepatic notch angle before (median: 126.3, IQR: 26.05) and after treatment (median: 136.9, IQR: 17.30), z = −1.783, *p* = 0.075.

### 3.8. Indicators of Portal Hypertension

Assessment of the signs of portal hypertension, such as enlargement of portal trunk, splenic vein, and superior mesenteric vein, and splenomegaly showed significant differences only for splenic volume before compared to after treatment (median: 564.79 cm^3^, IQR: 342.54 vs. median: 474.45 cm^3^, IQR: 330, z = −2.500, *p* = 0.012).

There were no differences between the portal trunk, splenic vein, and superior mesenteric vein diameter before and after treatment.

## 4. Discussion

During these past decades, cirrhosis has evolved from an irreversible liver disease into a potentially reversible one. As it has been convincingly demonstrated by recent studies, the reversibility of cirrhosis is no longer a myth, while results show significant improvement of architecture of the liver [6,8,9,24]. The main question is what happens after SVR with the liver: will the morphological changes remain as such, or will there be further morphological hepatic improvement?

Our study showed that there were some improved morphological aspects such as decrease of the liver and spleen volume, widening of the right hepatic vein diameter, increase of the sum of hepatic vein diameters and of Huber’s score. The first morphological improvement after treatment was the decrease in liver volume. Although the decrease in volume was statistically significant, it is small in value and therefore we consider that it is more likely secondary to the reduction of inflammation and may not represent a real loss of liver volume. The most dynamic changing parameter was the hepatic veins diameter, especially the right hepatic vein, significantly increased from a median of 6.36 mm at baseline to 8.12 mm (*p* < 0.001) after treatment. This variation has been also reported by other studies evaluating the fibrotic changes in pre-cirrhotic or cirrhotic patients [21]. We estimate that this parameter is the most sensitive one and can be used as an early marker of liver recovery. It is unclear whether this improvement is secondary to inflammation or it is a true indicator of fibrosis reduction. The Huber’s score also improved after treatment but only due to the change in the vessel diameter.

The widening of the periportal space consequently to atrophy in segment IV, as well as the caudate-right lobe ratio and its modified version, show no significant variation. A similar situation is the presence of the hepatic notch—the boundary between the hypertrophied caudate lobe and the right hepatic lobe—which also shows no improvement. We consider that the amplitude of the reversibility in fibrotic changes does not include these segment-volume variations. It is premature to set the boundaries of the reversibility in short-term follow up, as it could take longer to see a change in this parameter.

In the evaluation of the portal system vessel diameter variations we observed no statistically significant changes. This lack of modifications may reflect a balance between two opposite tendencies: one towards a reduction in diameter as a consequence of flow reduction and the other towards an enlargement due to portal hypertension. The same situation was noticed in other studies on cirrhotic patients [18]. The improvement of portal hypertension status was revealed by a decrease in splenic volume, suggesting that this could be a sensitive marker. The decreased splenic volume may be the result of a combination of factors: decreased portal hypertension and general inflammatory status, but further research is required.

Our study has a few strengths and several limitations. Thus, as far as we know, this is the first prospective study evaluating the morphological changes by CT in HCV-related cirrhosis patients achieving SVR after DAAs treatment. One of the limitations is the low number of patients included in the study. In addition, the one-dimensional measurements may not accurately reflect all intrahepatic morphological changes.

## 5. Conclusions

SVR in patients with HCV-related compensated cirrhosis treated with DAAs is associated with some improvements of hepatic morphology detectable by CT, the most constant being the increase of right hepatic vein diameter. These results are promising but should be validated by further studies based on a larger number of patients and a longer follow-up.

## Figures and Tables

**Table 1:** Liver morphological changes: pre- and post-treatment.

Variable	Pre-Treatment		Post-Treatment		*p*
	Median	IQR	Median	IQR	
Liver volume (cm^3^)	1786.77	879.23	1716.44	840.50	0.049
Caudate-right lobe ratio	0.65	0.19	0.65	0.17	ns
Modified caudate-right lobe ratio	0.98	0.28	0.99	0.31	ns
Right hepatic vein diameter (mm)	6.36	3.94	8.12	4.20	<0.001
Middle hepatic vein diameter (mm)	5.70	2.48	5.91	2.51	ns
Left hepatic vein diameter (mm)	6.69	3.17	6.65	3.19	ns
Sum of hepatic vein diameters (mm)	19.29	9.52	21.44	9.06	0.028
Huber’s score	19.16	11.50	20.58	9.73	0.035
Central periportal space widening (mm)	11.85	5.75	11.7	6.47	ns
Right posterior hepatic notch (degrees)	126.3	26.05	136.9	17.30	ns
Splenic volume (cm^3^)	564.79	342.54	474.45	330.00	0.012
Portal trunk diameter (mm)	14	2.70	14.2	2.90	ns
Superior mesenteric vein diameter (mm)	12.7	2.90	12.2	2.70	ns
Splenic vein diameter (mm)	9.85	3.31	10.2	3.73	ns

ns: nonsignificant; IQR: interquartile range.
